# Supplementary material for: Titanium dioxide dental implants surfaces related oxidative stress in bone remodeling: a systematic review
Source: PeerJ. 2022 Mar 3;10:e12951. doi: 10.7717/peerj.12951 (PMC8898546; doi:10.7717/peerj.12951)
Supplement: Supplemental Information 2 [file peerj-10-12951-s002.docx]

**Systematic Review and/or Meta-Analysis Rationale**

For systematic reviews, authors need to provide the following information:

1. The rationale for conducting the systematic review;

Dental implants are commonly used in dentistry, orthopedic surgery and other specialties that work on human skeleton. Biocompatibility properties of these materials have been studied extensively. However, there is debate over the function of ROS in the remodeling process and the dental implants' long-term stability. The key function of ROS in angiogenesis-osteogenesis coupling may influence the effectiveness of dental implant osseointegration because bone is a highly vascularized tissue (Hu *et al.*, 2018). There has recently been a growing body of evidence demonstrating the link between ROS generation during intraosseous dental implant implantation and bone vascularization and remodeling. The primary objective of this systematic review is to estimate the extent of ROS release after dental implant placement in bone and its impact on bone remodeling.

High quality and quantity of bone regeneration is the ultimate aim of an implanted biomaterial. Surgical trauma leading to inflammation is natural sequale during implantation procedure generating ROS that is necessary to drive multiple signal transduction for molecular healing process. Very often the site of implantation needing repair has already been infected and a certain degree of ROS production has already pre-existed even before surgical implantation of biomaterial. Combination of pre-existing ROS together with postoperative implantation trauma may produce an amount of oxidative stress that may exceed the antioxidant capacity in that particular site. This challenges may lead to inadequate bone implant integration and without further support with anti-inflammatory and antibiotics and adjustment of loading forces may lead to failure of osseointegration. However, the mechanism regulating the interaction between ROS and peri-implant environment with respect to producing ideal integration is poorly understood. Various molecular processes that interact with the biomaterial surface topography, peri-implant tissues, angiogenesis and antioxidants measures have been implicated in this systematic review.

1. The contribution that it makes to knowledge in light of previously published related reports, including other systematic reviews

This article is a conventional systematic review paper and because of the heterogenicity of the results, we could not perform meta-analysis.

This systematic review was conducted with the standard regulations of Preferred Reporting Items for Systematic Reviews and Meta-Analysis (PRISMA) statement.

1. Please ensure that statistical tests are reported in accordance with our guidelines

This systematic review lacking any statistical analysis because it is not meta-analysis.
